# Tailored Surgery in Inguinal Hernia Repair. The Role of Subarachnoid Anesthesia: A Retrospective Study

**DOI:** 10.1515/med-2019-0070

**Published:** 2019-10-29

**Authors:** Piergaspare Palumbo, Sofia Usai, Chiara Amatucci, Saverio Cerasari, Bruno Perotti, Luca Ruggeri, Roberto Cirocchi, Guglielmo Tellan

**Affiliations:** 1 Department of Surgical Sciences, “Sapienza” University of Rome, Rome, Italy; 2 Department of Emergency, Anesthesia and Critical Care, “Sapienza” University of Rome, Rome, Italy; 3 Department of Surgical Sciences, University of Perugia, Perugia, Italy

**Keywords:** Inguinal hernia repair, Subarachnoid anesthesia, Prilocaine

## Abstract

Safety and effectiveness evaluation of subarachnoid anesthesia implemented with hyperbaric Prilocaine in reduced dose (30mg) in combination with Fentanyl (20mcg), for the purpose of ensuring an optimal analgesia in open inguinal hernia repair.

Although the local anesthesia is the first line treatment for open inguinal hernia repair, a minority of patients is not eligible because of obesity or big groin hernia, requiring a high dose of local anesthetic. Subarachnoid anesthesia implemented with hyperbaric Prilocaine in reduced dose in combination with Fentanyl may be a good alternative.

Thirty patients were treated with intrathecal association of Prilocaine 30 mg and Fentanyl 20 mcg (group PF); they were compared to a group of fifty three ones, previously treated with a classic procedure with intrathecal Prilocaine 60 mg (group P).

The sensitive blockage remained within an higher limit at T12 level in the patients of PF group, and a lower limit at S1 level 50 minutes after the anesthesia, while in the P group the anesthetic tended to migrate (p<0.0001). In PF group 70 minutes after the anesthesia 21 patients had a Bromage score equal to 0 and 9 patients equal to 9 (in P group, 19 patients had a score equal to 3, 8 to 2 and 3 to 1, p<0.0001).

Subarachnoid anesthesia using Prilocaine 30 mg + Fentanyl 20 mcg could be stated as a viable alternative to local anesthesia in selected patients.

## 1 Introduction

Different surgical and anesthesiological techniques are used in inguinal hernia treatment. Most of the surgeons perform a “tailored surgery”, that is a kind of surgery adjusted on the single patient, both in relation to the surgical access (anterior, posterior, laparoscopic), both in relation to the kind of surgical and anesthesiological procedure carried out, and finally in relation to the prosthetic material chosen. In primitive unilateral inguinal hernia it would be good performing an open anterior access in local anesthesia [1,2]. European Hernia Society Guidelines indeed recommend to limit the use of subarachnoid anesthesia, particularly if long acting drugs or high doses of local anesthetics are used, because of the possible occurrence of complications, particularly urinary retention [1].

Furthermore, some patients (especially obese or overweight patients, or those affected by voluminous hernias) couldn’t be easily treated- managed under local anesthesia, since they could need high doses of anesthetics in order to obtain an acceptable analgesic result. That could lead to an increased risk of cardiac arrhythmia [3].Moreover, other patients prove to be poorly compliant to local anesthesia, fearing that they could feel pain during the surgical procedure. In these cases, general anesthesia could be useful, even if it is more invasive and could delay the discharge [4].

In this sense, subarachnoid anesthesia could represent an excellent option, provided that appropriate drugs and technique are employed. But in truth, the latest techniques in anesthesia, which provide for unilateral anesthetic blockage^5^ and low doses of short-acting drugs [6,7], enable to minimize disadvantages classically related to this kind of anesthetic technique and to achieve an earlier

recovery from motor and sensitive lower limb blockage. This allows a quicker discharge, particularly in patients admitted in Day Surgery facilities [8,9].

This study main endpoint is to evaluate safety and effectiveness of subarachnoid anesthesia with hyperbaric Prilocaine in reduced dose (30mg) in combination with Fentanyl (20mcg), for the purpose of ensuring an optimal analgesia. In several studies indeed the intrathecal administration of Fentanyl proved to enhance the anesthetic action so as to reduce considerably its dosage, without negative side-effects [10, 11, 12]. The secondary endpoint was to compare this technique with the traditional one, in order to analyze the recovery timing from the sensitive and motor blockage and to assess the opportunity to achieve an earlier and more effective postoperative recovery.

## 2 Materials and methods

Six-hundred fifty three patients underwent open anterior prosthetic hernioplasty in order to repair monolateral primitive inguinal hernia from January, 2016 to October, 2017, in Day and Week Surgery Unit of the Surgical Sciences Department – University of Rome “Sapienza”. Among this cohort, the patients eligible for subarachnoid anesthesia were selected. Inclusion criteria were: BMI ≤35 or inguinal defects size up to 3 cm. Patients with an age ≤18 years, ASA score III-IV, BMI ≤15 or >40, hepatic failure, acute kidney injury or chronic renal failure, heart failure, reported allergy to local anesthetics, congenital or acquired bleeding disorders were considered not eligible for the surgical and the anesthesiological procedure. Patients with an age ≥75 years were not excluded from the study, because, even if they could have worse general health status and higher ASA scores than the younger patients, the surgical morbidity results to be not significantly different [13].

Patients affected by bulky scrotal hernia were excluded, because, despite being eligible for subarachnoid anesthesia, they needed different techniques and prosthetic materials compared to the standard depicted above. Patients affected by severe diseases in cardiac conduction or by congenital or acquired methemoglobinemia were excluded, since the use of Prilocaine is contraindicated [14].

Finally, patients who denied consent to subarachnoid anesthesia were excluded.

A total of sixty cases were retrospectively selected, and patients were assessed considering two groups, a group PF (Prilocaine/Fentanyl), composed by 30 patients treated with intrathecal association of hyperbaric Prilocaine 30 mg and Fentanyl (Fentanest®- Pfizer) 20 mcg, and considered the object of the study. These patients were compared with a group P (Prilocaine), composed by 30 patients previously having surgery, and treated only with intrathecal hyperbaric Prilocaine (Prilotekal®- Molteni) 60 mg, and considered the control group.

The study has been taken into account as completed in his first testing phase to the achievement of 60 cases. Patients received subarachnoid anesthesia in seated position [15], with median access at the L3-L4 intervertebral space, using a Whitacre 24G spinal needle.Pin Prick test was performed in order to evaluate the sensitive block, while modified Bromage scale was used to assess the motor block at regular time intervals (at first 5 minutes and then 10 minutes until the end of surgical procedure). Blood pressure was recorded, and pain relief was evaluated through NRS scale at each interval time. In order to homogenize as much as possible the data subjects of evaluation in surgical procedure duration and algogenicity, open anterior prosthetic inguinal hernioplasty was carried out in all the patients. Open tension-free Lichtenstein repair of inguinal hernia with use of fibrin glue for mesh fixation was performed [16]. The hernia sac was dissected and sunk without its resection. No patients received a prophylactic antibiotic therapy, since it was not recommended by EHS guidelines [1]. In the postoperative time, the recovery from motor block, pain relief intensity, eventual painkiller administered, urinary voiding and the patients’ gait were evaluated. Sensitive block trend was monitored from the moment immediately following the intrathecal anesthetics administration until 80 minutes after the end of the surgical procedure. Equally, the motor block was assessed through Bromage Scale [17], the pain was scored through NRS (Numeric Rating Scale).

Discharge accuracy was determined through modified PADSS [18]. Lastly, data obtained were subjected to statistical analysis, and T-Student’s Test was used to compare age, weight, height, BMI, surgical procedure duration and discharge time. Non-parametric Mann Whitney test was used to compare blood pressure values, the motor block and the NRS score between the groups at T0 (before the surgical procedure) and during the postoperative period. Chi-square test was useful to compare the success variable in maintaining standing position, voiding and the administration of drugs in order to control pain. Analysis was performed through STATA 14.1 (StataCorp LLC). P-value <0.05 was considered statistically significant. The article has been edited in line with the STROCSS criteria, following the STROCSS guidelines.

Ethical approval: The study did not use experimental drugs or procedures, and obtained the Department Scientific Committee approval. The consent to publish from the participant (or legal parent or guardian for children) to report individual patient data was obtained.

## 3 Results

The results obtained are reported in Table 1. The two groups do not differ with regards to age, weight, height and BMI. All the procedures were performed by the same anesthesiological equipe, who monitored the patients during the surgical procedures and the post-operative period. The mean duration of subarachnoid anesthesia procedure from the patient positioning to the anesthetics injection results to be comparable between the two groups, and it stands at about 5 minutes. The mean surgical procedure time was 53.7 minutes (SD=5.6) in the group P and 55.3 (SD=14.8) in the group PF (p-value=0.566, Table 1.).No surgical intraoperative complications or procedural difficulties occurred, no anatomical anomalies were reported.

**Table 1:** Anesthetic blockage trend.

INTRAOPERATIVE ASSESSMENT	GROUP PF (Prilocaine 30mg+Fentanil 20mcg)	GROUP P (Prilocaine 60mg)	
SURGICAL PROCEDURE	75.3min (SD=14.8)	73.7 min (SD=5.6)	p=0.566
HIGHER LIMIT SENSORIAL BLOCK			
- 3 min	26 patients from T12	26 patients from T12	
	4 patients from T11	4 patients from T11	
- 5 min	30 patients from T12	19 patients from T12	
		10 patients from T11	
		1 patient from T10	
- 5-50 min	invariate	invariate	
- 60 min	invariate	20 patients from T12	
		10 patients from T11	
- 70 min	invariate	1 patient from T12	
		21 patients from L1	
		8 patients from L2	
- 80 min	invariate	8 patients from L1	
		22 patients from L2	
LOWER LIMIT SENSORIAL BLOCK			
- 3 min	27 patients at S2	28 patients at S2	
	3 patients at S3	2 patients at S3	
- 5 min	invariate	15 patients at S2	
		14 patients at S3	
		1 patient at S4	
- 10 min	1 patient at S1	invariate	
	29 patients at S2		
- 20 min	30 patients at S2	invariate	
- 30-50 min	30 patients at S1	invariate	
- 60 min	7 patients at S4	3 patients at S1	
	23 patients at S5	17 patients at S2	
		10 patients at S3	
	23 patients at S5	17 patients at S2	
		10 patients at S3	
- 70 min	30 patients at L3	3 patients at L5	
		5 patients at S1	
		19 patients at S2	
- 80 min	21 patients at L2	3 patients at S3 12	
	9 patients at L3	12 patients at L4	
		17 patients at L5	
		1 patient at S1	
MOTOR BLOCK (Bromage Scale) - 3-5 min			
- 3-5 min	30 patients 3	30 patients 3	
- 10 min	6 patients 3	30 patients 3	p<0.001
	24 patients 2		
- 20 min	30 patients 2	30 patients 3	p<0.001
- 30-50 min	30 patients 1	30 patients 3	p<0.001
- 60 min	30 patients 1	19 patients 3	
		11 patients 2	p<0.001
- 70 min	9 patients 1	19 patients 3	
	21 patients 0	8 patients 2	
		3 patients 1	p<0.001
- 80 min	30 patients 0	5 patients 3	
		21 patients 2	
		1 patient 1	
		3 patients 0	

Blood pressure monitoring throughout the surgical procedure pointed out a satisfactory heamodynamic stability, without significant differences between the groups.

Group PF: At 3 minutes, 26 patients showed a motor block from T12 and 4 patients from T11; at 5 minutes, this block settled down T12 in all patients and it remained stable until 80 minutes. The lower limit of sensitive block at 3 minute was at the level of S2 in 27 patients and of S3 in 3 patients, remained unchanged in the 5 minutes-evaluation. At 10 minutes, 29 patients presented a sensitive block at the level of S2 and an only patient at the level of S1; this level was constant until the 50 minutes evaluation from the anesthetics injection; particularly, all the patients were aligned on S2 level after 20 minutes and on S1 level after 30 minutes.

In the 60 minutes evaluation, 7 patients showed the sensitive block at S4 level and 23 patients at S5 level, going back at L3 level in the 70 minutes evaluation. In the final 80 minutes assessment, 21 patients presented a lower blockage limit on L2 and 9 patients on L3. With regards to the motor blockage, all patients in the range from 3 to 5 minutes obtained a score equal to 3; at 10 minutes, 24 patients had a score equal to 2 and 6 patients equal to 3. At 30 minutes, all patients reached a score equal to 1, which maintained constant until 60 minutes. In the 70 minutes evaluation, 21 patients had a score equal to 0 and only 9 patients remained stable with a score equal to 1. At 80 minutes, all patients had a score equal to 0. Finally, pain relief was evaluated at the respective time intervals during the surgical procedure (Table 1). All patients were informed as regards the difference in touch and pain relief, so as to easily report the latter through NRS scale. In the 3 minutes evaluation, all patients reported pain equal to 1, while at 5 minutes 7 patients (25%) equal to 0 and the outstanding 23 patients (75%) equal to 1 (p<0.001). From 10 to 50 minutes, all patients reported pain equal to 0. At 60 minutes, 11 patients reported pain equal to 0 and 19 patients equal to 1. In the last two assessments, respectively at 70 and 80 minutes, all patients reported pain with a value equal to 1 (Table 2). The results of patients follows-up were, finally, analyzed, once they left the operating theatre and got back to their ward. The sensitive blockage in the immediate post-operative time, had an extension equal to L1-L3 in 9 patients (30%) and to L1-L4 in the remaining 21 (70%). Moreover, in the 30 minutes-evaluation after they returned in their ward, all patients had already recovered from the voiding inhibition and could regain the standing position; moreover, nobody needed supplementary drugs administration to control pain (Table 3). In the subsequent evaluation at 60 minutes, all patients reached the standing position, 5 patients needed the administration of drug to control pain (17%). The mean time to discharge (after adequate postoperative observation) was 185,4 minutes (SD=7.9).

**Table 2:** Intraoperative pain relief assessment.

INTRAOPERATIVE ASSESSMENT	GROUP PF (Prilocaine 30mg+Fentanil 20mcg)		GROUP P (Prilocaine 60mg)	
DOLORE NRS				
- 3 min	30 patients 1		30 patients 1	
- 5 min	7 patients 0		30 patients 0	p<0.001
	21 patients 1			
- 10-50 min	30 patients 0		30 patients 0	
- 60 min	11 patients 0		6 patients 0	
	19 patients 1		11 patients 1	
			13 patients 2	p=0.01
- 70 min	30 patients 1		13 patients 1	
			8 patients 2	
			9 patients 3	p=0.001
- 80 min	30 patients 1		4 patients 2	
			14 patients 3	
			9 patients 4	
			3 patients 5	

**Table 3:** Post-operative assessment (sensorial and motor blockages, voiding, standing position, need for pain management).

POST-OPERATIVE ASSESSMENT	GROUP PF (Prilocaine 30mg+Fentanil 20mcg)		GROUP P (Prilocaine 60mg)
SENSORIAL BLOCK			
- 30 min after the end of surgical procedure	9 patients L1-L3	8 patients L1-L4	
	21 patients L2-L4	14 patients L2-L5	
		4 patients L2-L4	
		4 patients L2-L5	
MOTOR BLOCK (Bromage Scale)			
-30 min after the end of surgical procedure	30 patients 0	4 patients 0	
		18 patients 1	
		8 patients 2	p<0.001
- 60 min after the end of surgical procedure	invariate	30 patients 0	
VOIDING			
- 30 min after the end of surgical procedure	30 patients	0 patients	
- 60 min after the end of surgical procedure	30 patients	19 patients	
STANDING POSITION			
- 30 min after the end of surgical procedure	30 patients	0 patients	
- 60 min after the end of surgical procedure	30 patients	20 patients	
NEED FOR PAIN MANAGEMENT			
- 30 min after the end of surgical procedure	0 patients	15 patients	p<0.001
- 60 min after the end of surgical procedure	5 patients	20 patients	p0<0.001

Group P: In the 3 minutes-evaluation, 26 patients showed a sensitive blockage extended from T12 while in 4 patients it was extended from T11; at 5 minutes, this blockage was stable at the level of T12 in 19 patients, T11 in 10 patients and T10 in an only patient, remaining unchanged until 50 minutes. In the 60 minutes-evaluation, patients with T12 blockage were increased up to 20 and those with T11 blockage up to 10, while in the subsequent assessment at 70 minutes, an only patient showed the blockage at T12 level, 21 patients at L1 and 8 patients at L2. Finally, in the 80 minutes-evaluation, 8 patients had a L1-level blockage and 22 patients a L2-level. The lower limit of this sensitive blockage, at 3 minutes was at S2 level in 28 patients and at S3 level in 2 patients, while at 5 minutes this level was confirmed at S2 level in 15 patients, S3 level in 14 patients and migrated at S4 level in 1 patients, remained unchanged in the subsequent evaluations until 50 minutes. At the 60 minutes-evaluation, 3 patients showed the blockage at S1 level, 17 patients at S2 and 10 patients at S3; at 70 minutes, the blockage lower limit migrated at L5 level in 3 patients, S1 level in 5 patients, S2 level in 19 patients and S3 level in 3 patients. Finally, in the last 80 minutes-evaluation, 12 patients presented the blockage at L4 level, 17 at L5 level and an only patient at S1 level. The motor blockage development showed that all patients at 3 minutes reached a score equal to 3, maintaining constant in the subsequent assessments until 50 minutes. At the 60 minutes-evaluation, this score was equal to 3 in 19 patients and equal to 2 in 11 patients; at 70 minutes, it was equal to 3 in 19 patients, equal to 2 in 8 patients and equal to 1 in 3 patients. In the last 80-minutes assessment, 5 patients reached a score equal to 3, 21 patients equal to 2 and an only patient equal to 1, while only 3 patients reached a score equal to 0.

All patients reported pain equal to 1 at 3 minutes, while at 5 minutes all patients agreed upon a score equal to 0, which remained constant in all the subsequent assessments until 50 minutes. In the 60 minutes-evaluation, 6 patients reported a pain score equal to 0, 11 patients a score equal to 1 and 13 a score equal to 2. At 70 minutes, 13 patients reported a NRS score equal to 1, 8 patients equal to 2 and 9 patients equal to 3. In the last 80-minutes assessment, 4 patients stated a NRS score equal to 2, 14 patients a score equal to 3, 9 patients equal to 4 and 3 patients equal to 5 (p value<0.001).

Finally, the patients follow-up results were analyzed, once they left the operating theatre and got back to their ward.

The sensitive blockage in the immediate postoperative period had an extension from L1 to L4 in 8 patients (27%), from L1 to L5 in 14 patients (47%), from L2 to L4 in 4 patients (13%) and from L2 to L5 in 4 patients (13%). The motor blockage score resulted equal to 0 on Bromage Scale in only 4 patients (13%), while 18 patients obtained a score equal to 1 and 2 patients equal to 2 (p<0.001). In the 60 minutes evaluation, all the patients were aligned upon a score equal to 0 on Bromage Scale. At 30 minutes, nobody amongst the group P patients recovered from voiding inhibition, nobody could regain the standing position and 15 patients needed the administration of drugs in order to control pain. In the subsequent evaluation at 60 minutes, only 19 patients regained voiding (63%), 20 patients regained the standing position (66%), 20 patients needed the additional administration of drugs to control pain (66%). A patient needed bladder catheterization. The mean discharge time was about 215.6 minutes (SD=7.9).

## 4 Discussion

International clinical practice guidelines recommend to limit the use of subarachnoid anesthesia in patients submitted to inguinal hernioplasty, suggesting the local anesthesia as the first line treatment [1,2].

However, the improvement of the regional anesthesia technique with the use of short acting anesthetic agents has made more suitable this practice, particularly in selected cases, detailed for obese patients, large inguinal defect (>3 cm) and scrotal hernias, in which high volumes of anesthetic are required. All patients have been assessed by the PADSS system [18], paying special attention to early ambulation, to ease urination, absence of nausea and vomiting, and the severity of pain. Each item can reach a score ranging from 0 and 2 and the discharge is possible with a score ≥ 9. Nevertheless, even a single item equal to 0 does not allow the quick discharge and requires an admission in a long-stay ward.

Thus, it is clear that the success of the clinical pathway is related to a good control of patients’ mobility.

The choice in using Prilocaine added with Fentanyl is supported by a large number of clinical evidences, whereby an opioid (drug) added to a local anesthetic could improve the effectiveness of intraoperative analgesia and extend the length of the postoperative one, due to an analgesic synergy between the intrathecal opioid administration and the local anesthetic, with a dose-dependent length of action [19, 20, 21]. The synergy is strictly related to opioid and local anesthetic pharmacodynamics, both acting on presynaptic calcium receptors by inhibiting the neurotransmitters relapse. Fentanyl, used alone, increases the potassium channels conductance, while Prilocaine blocks calcium channels, facilitates the postsynaptic hyperpolarization, and therefore inhibits the nerve transmission [22, 23, 24, 25].

Main benefits of using a Fentanyl-Prilocaine combination in the management of patients in Day Surgery

are represented by an enhancement of the sensory block without lengthening the motor block.

The motor block is indeed the main obstacle to early discharge, hindering the ability of ambulation and the restoration of the spontaneous urinary function [26,27].The assessment of the upper and lower limit sensory block has highlighted the tendence of the block to be confined in the chose dermatomer in the patients included in PF group, treated with Fentanyl-Prilocaine combination, unlike patients included in P group, treated only with Prilocaine, in which the anesthetic tends to spread in craniocaudal side more rapidly.

This effect is due, at first, to smaller volume of given Prilocaine, but it is not possible to exclude the bariatric properties of fentanyl, affecting the local anesthetic spreading.

The so obtained block appears to be selective on dermatomers interested by surgical intervention, with clear benefits for the patients’ management. Furthermore, Fentanyl improves the effect of the local anesthetic and makes it possible to obtain an effective analgesia in the postoperative period, having a safe pharmacologic effect and side-effects free, by limiting further administration of analgesic agents.

The disposal of the motor block, in the PF group, is effective in the space of the operation time, in the PF group, as confirmed by our data. Seventy minutes after anesthesia 21 patients reached a Bromage score equal to 0, and 9 equal to 1, and everyone was able to move itself from the operating table to the stretcher. All the patients willingly voided within 60 minutes from the end of surgical procedure.

By contrast, in the P group, 19 patients reached a score equal to 3, 8 of them equal to 2, and only 1 reached a score equal to 1.

A timely resumption of the urination is one of the main benefits obtained by the limited dermatomer block, due to the swift regression (backward step) to a level up to S2.

A review of the collected data emphasizes that all the patients of the PF group, 70 minutes after anesthesia, were estimated to have a block at L3 level, while, at the same time, in the P group, only 8 cases had a block over the S1 level, and even 22 had a level block between S2 and S3, with evident delay of the resumption of spontaneous urination.A further scoring item useful for the assessment of the correct discharge time, is the evidence of nausea or vomiting. None of the subjects in the both groups reported PONV, and everyone reached the highest score.

This therefore confirms that the subarachnoid anesthesia using Fentanyl, doesn’t increase a risk of nausea [28].

All the patients, lastly, evidenced a higher “pain free” level during the central time of the operation.

The following assessment carried out 70 minutes after the anesthesia revealed, in the PF group, a NRS score of 1 in all patients, without the need of further analgesics; in The P group, instead, 13 patients referred a pain score equal to 1, 8 equal to 2, and 9 equal to 3. In the final assessment at 80 minutes, pain occurred in 4 patients with a score of 2, in 14 with a score of 3; 9 patients referred pain score equal to 4, and 3 equal to 5: In the last two groups the administration of paracetamol was required.

A better analgesic control, therefore, has been obtained by the Prilocaine + Fentanyl combination.

With regard to the total admission time, the duration of the surgical time is similar in both groups: 73.7 minutes (SD= 5.6) in the P group and 75.3 (SD= 14.8) in the PF group (p=0.566).

The stay time in the postoperative ward before discharge, by contrast, was 21 minutes shorter in the PF group, highlighting a more rapid recovery in the patients’ group given Prilocaine+Fentanyl.

We believe that regional subarachnoid anesthesia using Prilocaine 30 mg +Fentanyl 20 mcg could be stated as a viable alternative to local anesthesia in selected patients undergoing inguinal hernia repair. A suitable position of the patient carrying out the selective anesthesia is compulsory. The operative field is free from anesthetic liquid excess and the muscle and fascia plans appears more clearly identified, particularly in obese patients. The opioids addition in the anesthetic blend is safe and effective and reduces the use of analgesics in the postoperative time, without the inconvenience of the urinary retention. The limitation of this study is the small size of the sample considered, since being a first testing phase, so that further studies are needed.

The datasets used and analyzed during the current study are available from the corresponding author on reasonable request.The authors declare not to have competing interests. Acknowledgements – not applicable.
